# Patient-derived xenograft models capture genomic heterogeneity in endometrial cancer

**DOI:** 10.1186/s13073-021-00990-z

**Published:** 2022-01-10

**Authors:** Vanessa F. Bonazzi, Olga Kondrashova, Deborah Smith, Katia Nones, Asmerom T. Sengal, Robert Ju, Leisl M. Packer, Lambros T. Koufariotis, Stephen H. Kazakoff, Aimee L. Davidson, Priya Ramarao-Milne, Vanessa Lakis, Felicity Newell, Rebecca Rogers, Claire Davies, James Nicklin, Andrea Garrett, Naven Chetty, Lewis Perrin, John V. Pearson, Ann-Marie Patch, Nicola Waddell, Pamela M. Pollock

**Affiliations:** 1 School of Biomedical Sciences, Queensland University of Technology located at the Translational Research Institute, Brisbane, QLD Australia; 2 The University of Queensland Diamantina Institute, The University of Queensland, Woolloongabba, QLD Australia; 3 Department of Genetics and Computational Biology, QIMR Berghofer Medical Research Institute, Brisbane, QLD Australia; 4 Mater Health Services, South Brisbane, QLD Australia; 5 Mater Pathology, Mater Research, Brisbane, QLD Australia; 6 The University of Queensland, Brisbane, QLD Australia; 7 The Wesley Hospital, Auchenflower, QLD Australia; 8 Icon Cancer Centre Wesley, Auchenflower, QLD Australia

**Keywords:** Endometrial cancer, Patient-derived xenografts, Genomic characterization, Mutational signatures, Genomic scarring, Tumor heterogeneity, Homologous recombination, PARP inhibitors

## Abstract

**Background:**

Endometrial cancer (EC) is a major gynecological cancer with increasing incidence. It comprises four molecular subtypes with differing etiology, prognoses, and responses to chemotherapy. In the future, clinical trials testing new single agents or combination therapies will be targeted to the molecular subtype most likely to respond. As pre-clinical models that faithfully represent the molecular subtypes of EC are urgently needed, we sought to develop and characterize a panel of novel EC patient-derived xenograft (PDX) models.

**Methods:**

Here, we report whole exome or whole genome sequencing of 11 PDX models and their matched primary tumor. Analysis of multiple PDX lineages and passages was performed to study tumor heterogeneity across lineages and/or passages. Based on recent reports of frequent defects in the homologous recombination (HR) pathway in EC, we assessed mutational signatures and HR deficiency scores and correlated these with in vivo responses to the PARP inhibitor (PARPi) talazoparib in six PDXs representing the copy number high/p53-mutant and mismatch-repair deficient molecular subtypes of EC.

**Results:**

PDX models were successfully generated from grade 2/3 tumors, including three uterine carcinosarcomas. The models showed similar histomorphology to the primary tumors and represented all four molecular subtypes of EC, including five mismatch-repair deficient models. The different PDX lineages showed a wide range of inter-tumor and intra-tumor heterogeneity. However, for most PDX models, one arm recapitulated the molecular landscape of the primary tumor without major genomic drift. An in vivo response to talazoparib was detected in four copy number high models. Two models (carcinosarcomas) showed a response consistent with stable disease and two models (one copy number high serous EC and another carcinosarcoma) showed significant tumor growth inhibition, albeit one consistent with progressive disease; however, all lacked the HR deficiency genomic signature.

**Conclusions:**

EC PDX models represent the four molecular subtypes of disease and can capture intra-tumor heterogeneity of the original primary tumor. PDXs of the copy number high molecular subtype showed sensitivity to PARPi; however, deeper and more durable responses will likely require combination of PARPi with other agents.

**Supplementary Information:**

The online version contains supplementary material available at 10.1186/s13073-021-00990-z.

## Background

Endometrial cancer (EC) is the most common gynecological malignancy in developed countries with increasing annual rates [1]. While most ECs are detected early and have good prognosis, patients with metastatic disease (15%) or who relapse after surgery (~15%) have a median survival of less than 12 months [2].

EC is comprised of multiple histological subtypes, including low- and high-grade endometroid, serous, clear cell, and uterine carcinosarcomas. The Cancer Genome Atlas (TCGA) identified four molecular subtypes: *POLE* mutant with an excellent prognosis; MSI/”hypermutated” equivalent to mismatch repair deficient (MMRd) with an intermediate prognosis; copy number high (CN-high) or frequently *TP53* mutated (p53mut) with the worst prognosis; and copy number low (CN-low) or p53 wild-type with intermediate prognosis [3]. Diagnostic algorithms utilizing surrogate immunohistochemistry stains have since been adopted, with the CN-high subtype now identified as p53mut/p53-abnormal, the MSI subtype identified as MMRd, and POLE defined as only those samples with mutations in the *POLE* exonuclease domain. Similar algorithms have been developed by multiple labs and shown to be prognostically important in low-, intermediate-, and high-risk ECs [2, 4–6]. Genomic studies of uterine carcinosarcomas (UCS) have also revealed the presence of similar subtypes; however, the majority of tumors (~90%) contain *TP53* mutations and a low tumor mutation burden (TMB) [7–9].

Apart from the recent approval of immune checkpoint inhibitors (ICIs) for MMRd cancers, there has been little development in terms of precision medicine for EC. Surgery, radiotherapy, and chemotherapy still remain the main treatment options. In recent years, there has been a growing interest in applying PARP inhibitors (PARPi) for treatment of EC. PARPi have proven to be incredibly effective in cancers with homologous recombination (HR) deficiency, such as ovarian and breast cancers with *BRCA1/2* mutations. In EC, PARPi sensitivity was originally associated with PTEN loss [10], which has since been refuted using a larger panel of cell lines [11], as well as loss of MRE11 [12]. Mutations in *ARID1A*, which commonly occur in EC, have also been associated with PARPi sensitivity in cell lines from other cancers [13]. In 2017, a pan-cancer analysis of 102 HR DNA repair genes revealed that 2.4% of ECs have a combination of germline or somatic bi-allelic mutations and/or LOH [14]. In 2019, a BRCA-like genomic scar and COSMIC Signature 3 were reported in 41% and 46% of non-endometrioid EC TCGA cases (~90% p53mut), respectively [15].

In EC, there are very few cell lines derived from UCS and serous ECs. Multiple cell lines from endometrioid ECs carry *TP53* mutations; however, all of these cell lines are also MMRd and show a high TMB, suggesting that *TP53* mutations are acquired through culturing. Therefore, these cell lines are unsuitable as models for the poor prognosis CN-high/p53mut subtype. Identification of effective therapies and predictive biomarkers for CN-high/p53mut ECs requires well-characterized pre-clinical models that recapitulate this molecular subtype. Patient-derived xenografts (PDX) have been previously demonstrated as reliable pre-clinical models for assessing treatment responses, if carefully characterized. In this study, we performed in-depth genomic characterization of EC PDX models to define their suitability as pre-clinical models and predict HR deficiency status by assessing genomic scars. Here, we report in vivo responses to the potent PARPi talazoparib in a panel of PDX models representing the CN-high/p53mut and MMRd subtypes and correlate these responses with genomic features.

## Methods

### Patient samples

In this study, fresh surgical samples were obtained from 54 patients undergoing surgery for EC at the Mater public and private hospitals (*n*=49), Queensland, Australia, or the Wesley Private Hospital (*n*=5), Queensland, Australia, between May 2015 and February 2018. All patients provided written informed consent. The study has human ethics approval from the Mater Health Services Human Research Ethics Committee (HREC/15/MHS/127), UnitingCare Health Human Research Ethics Committee (1116), Queensland University of Technology (1500000169, 1500000323), and QIMR Berghofer (P3478, P2095). Clinical data including tumor stage, grade, chemotherapy treatment, and survival status was collected (Additional File 1: Table S1). The cohort had a median age of 70 years (range 43–86 years). Fresh tissue was obtained at surgery and transported to the laboratory on ice in RPMI, 10% FBS. The remainder was fixed in formalin and embedded in paraffin (FFPE). Where possible, a blood sample was also obtained for sequencing analysis. Outcome analysis was performed using date of initial diagnosis of primary disease and date of death due to EC.

### Mouse PDX models

PDX establishment and passaging was performed according to animal ethics approvals at TRI (TRI/021/19) and QUT (1900000701). Fresh primary tumors (*n*=33) were transplanted into immunocompromised Nod Scid Gamma (NSG) mice within 4 h of surgery. When transplantation could not be performed immediately (due to unavailability of mice or surgery occurring late in the day), tumors were either stored at 4 °C overnight in transport media (*n*=9) or viably frozen in 90% FBS and 10% DMSO (*n*=11). Each sample was cut into approximately 1–2 mm^3^ pieces and placed on ice in a 1:1 solution of RPMI:Matrigel. Mice were anaesthetized with isofluorane (induction 4%, maintenance 1.5%) and a single tumor piece was inserted subcutaneously in the subscapular region (2–4 mice). Mice were injected (26-G needle) prior to surgery with 0.075mg/kg Buprenorphine for pain management. PDX engraftment was then assessed weekly using micro-calipers. Once a tumor reached a volume of ~750–1000mm^3^, mice were euthanized using CO_2_ and several 1–2-mm^3^ fragments were transplanted subcutaneously into the next generation of mice. Mice were housed up to five/cage and standard laboratory chow was provided ad libitum. At passaging, a slice of each PDX was preserved as FFPE, as well as frozen for DNA extraction. Hematoxylin and eosin (H&E) slides were examined by an anatomical pathologist to determine the histology of each PDX passage and original patient tumor.

### In vivo drug testing

In vivo drug studies were performed according to animal ethics approvals at TRI and QUT (QUT/275/17 and 1700000755). Mice were implanted with PDX fragments from 6 to 10 weeks of age. Once tumors reached ~150–350 mm^3^ (faster models started drug between 150 and 250 mm^3^ and slower models between 250 and 350 mm^3^), mice were randomized into treatment groups and treated for 28 days via oral gavage. Final tumor volume was measured on day 29, 24 h after last dose. Drugging of each PDX model occurred over 2–3 passages with a similar number of mice in each arm from each passage. Average starting tumor volumes in vehicle and talazoparib-treated arms were similar and are listed below: PDX03, 275mm^3^ in both arms; PDX12, 167 and 163mm^3^, respectively; PDX23, 199 and 184mm^3^, respectively; PDX53, 205 and 223mm^3^, respectively; PDX49, 271 and 242mm^3^, respectively; and PDX56, 222 and 268mm^3^, respectively. Mice were drugged 6 days on/1 day off with vehicle (20% Tween 20, 20% DMSO) or talazoparib (0.33mg/kg) as previously reported [16].

### DNA extraction and quality control

DNA was extracted from patient blood samples as well as patient and related PDX tumor samples, using DNeasy Blood & Tissue Kits (Qiagen, Germantown, MD, USA). The purity of DNA was assessed using NanoDrop and quantified using the Qubit dsDNA BR assay (Thermo Fisher Scientific, MA, USA). DNA samples were assayed with the Omni 2.5-8, V1.0 and V1.1 Illumina BeadChip as per the manufacturer’s instructions (Illumina, San Diego, CA, USA). SNP array analysis was performed to confirm sample identity and tumor content of DNA samples [17] for subsequent sequencing, and is described in detail in the Additional File 1: Supplementary Material.

### Whole exome and whole genome sequencing

Eight PDX models underwent whole-exome sequencing (WES) and three p53-mutant PDX models underwent whole-genome sequencing (WGS). The WES libraries were prepared from 1 μg of genomic DNA using the SureSelect capture V5+UTR kit (Agilent, Santa Clara, CA, USA) and sequenced with 100bp paired-end sequencing on a HiSeq 2500/4000 (Illumina) to a targeted 100-fold read depth. The WGS libraries were prepared from 2 μg of genomic DNA using the TruSeq Nano kit (Illumina) and sequenced with 150bp paired-end sequencing on a HiSeq X Ten (Illumina) at Macrogen (Geumcheon-gu, Seoul, South Korea) with targeted mean read depth of 60-fold for primary tumor samples and 30-fold for matched PDX and normal samples.

### Sequencing data analysis

Cutadapt (v1.18) [18] was used to trim low-quality 3′ bases (“-q 20”) and remove adapters before alignment to a combined human/mouse (GRCh37/GRCm38 Nodshiltj background) reference using BWA-mem (v0.7.15) [19], and sorted and indexed using SAMtools (v1.9) [20]. Duplicate reads were marked using Picard MarkDuplicates (v1.97). Human mutation calling process used only read-pairs aligned to the human sequences with a mapping quality score of 60. Quality assessment and coverage estimation was carried out by in-house developed tools, qProfiler and qCoverage. Downstream analysis included variant calling, copy number alteration (CNA) and structural variant (SV) detection [21–23], heterogeneity analysis [24, 25], microsatellite instability (MSI) and HR deficiency (HRD) status assessment [26–28], and signature analysis [29, 30], described in detail in the Additional File 1: Supplementary Material.

Publicly available datasets (TCGA uterine corpus endometrial carcinoma (UCEC) and TCGA-UCS) were processed using a similar approach, without the initial human/mouse alignment and filtering step. For TCGA-UCEC and TCGA-UCS, only samples with >50 somatic substitutions were selected for the analysis (*n*=591; 5 samples excluded) to ensure accurate estimation of mutational signatures.

### Statistical analysis and data visualization

Statistical analysis and data visualization was performed in R 3.5.1, using ggplot2 and ComplexHeatmap packages, and using Circos. Final figure formatting was done with Illustrator (Adobe).

## Results

### Established PDX models represent four histological subtypes

Of 32 EC tumors implanted fresh, we generated 13 EC PDXs which were confirmed as EC models by a specialized anatomical pathologist. Successful engraftment rates were only obtained for histological grade 2 and 3 tumors implanted fresh (33 and 61%, respectively); none of grade 1 tumors engrafted (0/8) (Additional File 1: Table S2). We were also able to obtain 5 EC PDX models from an additional 22 EC tumors after storage at −80 °C or 4 °C overnight (Additional File 1: Table S2). In addition to the 18 successful EC PDXs mentioned above, seven patient tumor transplants showed in vivo growth; however, these were confirmed to be lymphomas based on positive leukocyte common antigen staining. The lymphomas occurred more often from grade 1 tumors (3/14, 21%) than grade 3 ECs (3/29, 10%) with an overall rate of 13% (7/54). The ability to develop a PDX model from engrafted tumors was significantly associated with shorter disease specific survival (*p* < 0.02) (Additional File 1: Fig. S1). This study reports detailed genomics data for 11 of these 18 EC PDX models based on the availability of DNA from matched blood and multiple passages at study commencement.

**Fig. 1:**
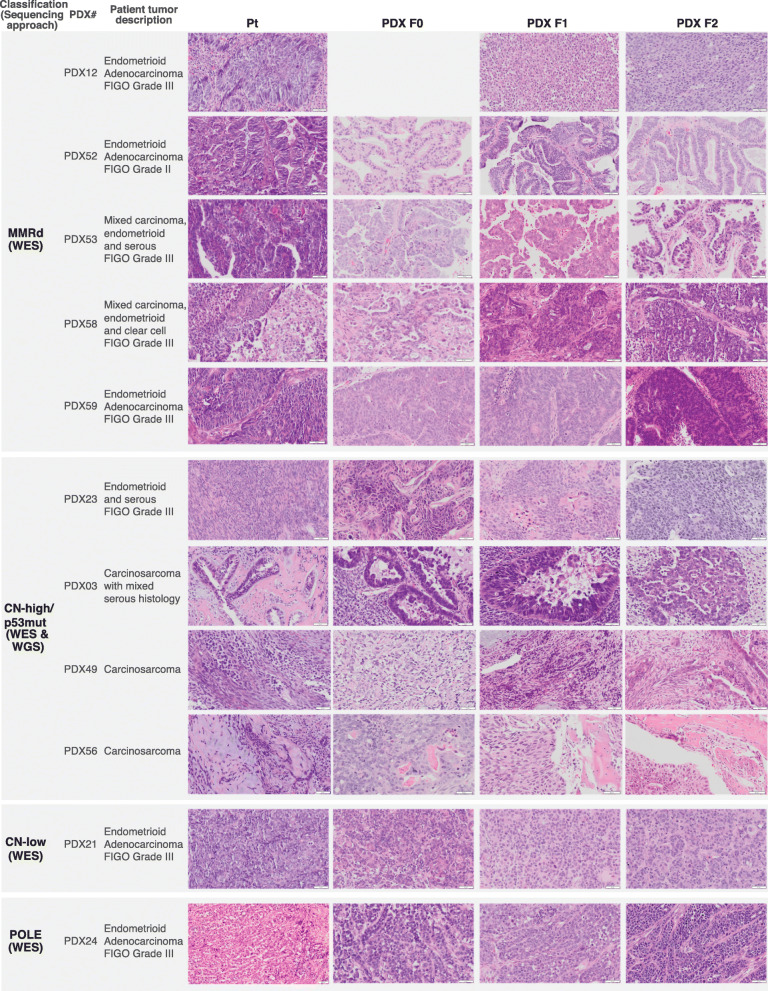
Histopathology assessment of the primary and PDX tumor samples. Pt, primary tumor. PDX F0, 1^st^ tumor obtained from the mouse transplant. PDX F1, PDX F2, subsequent transplants. F0 picture for PDX12 is missing as no FFPE sample was available for this lineage. Scale bars denote 50 μm, except for PDX24 Pt – 20 μm

The 11 PDX models were from EC of patients with a mean age of 70 (range 43–86 years, Additional File 1: Table S1) who represented a wide range of EC disease with varying histologies and stages (IA to IIIB). Histologic diagnoses included carcinosarcoma (*n*=3), mixed endometrioid and serous (*n*=2), mixed endometrioid and clear cell (*n*=1), and endometrioid (*n*=5, of which 4 were FIGO grade 3 and 1 FIGO grade 2). Nine patients received radiation or chemoradiation and six patients recurred, five of which have subsequently died. In all models, the tissue architecture, the epithelial compartment and the global histological classification features were preserved in the corresponding F0 to F2 PDXs (Fig. 1).

### Genomics of EC PDX models

**Fig. 2:**
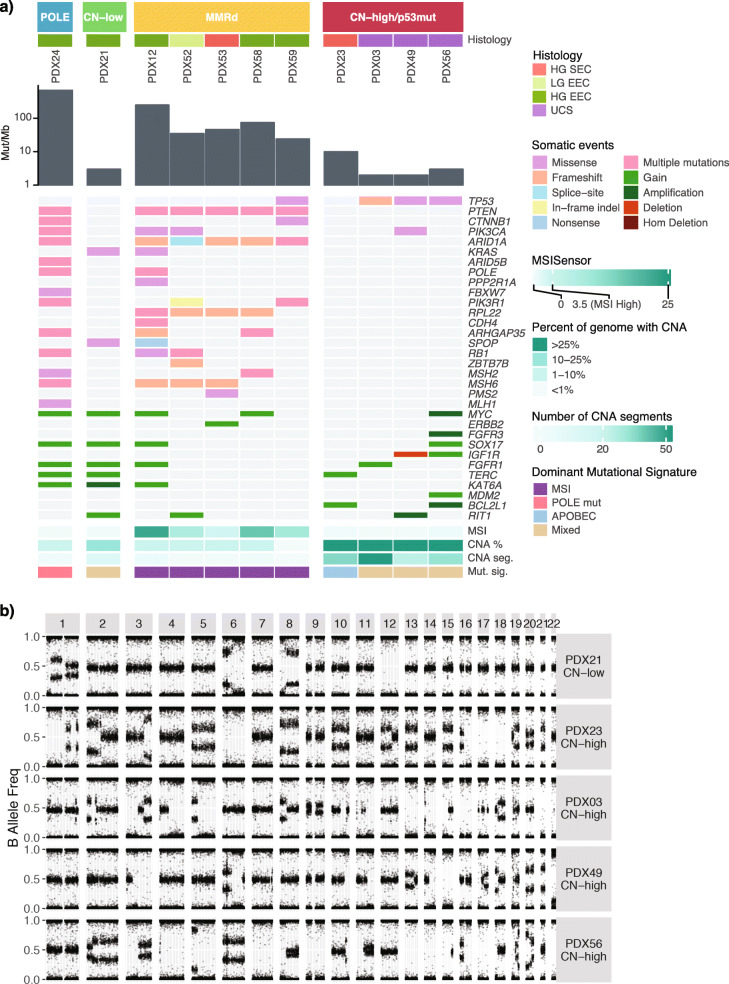
The four molecular subtypes are represented in PDX models. **A** Genomic characteristics of endometrial carcinoma and carcinosarcoma PDX models. PDX models are grouped by the four molecular subtypes: POLE, CN-low, MMRd, and CN-high/p53mut. Tumor mutation burden is shown by gray bars, as mutations per Mb using a log10 scale. Somatic mutations and CNA events, which were detected in PDX samples in MMR genes and genes relevant to endometrial carcinomas and carcinosarcomas (Additional File 1: Table S3, Additional File 2: Table S4), are shown. Only consensus variants detected in all sequenced PDX tumor samples were included in this figure. MSI score was assessed by MSISensor (Additional File 1: Fig. S3). Percentage of genome with CNA and the number of CNA segments were determined from SNP arrays or WGS data (Additional File 1: Fig. S4). Only the dominant mutational signature etiology is shown. **B** Representative B-allele frequency plots of CN-low and CN-high PDX models

Sequencing and SNP array analysis was performed to molecularly classify the 11 EC PDX models. The molecular classification was adapted from the TCGA endometrial and UCS studies and was based on five aspects (Fig. 2A, Additional File 1: Fig. S2): commonly mutated genes (Additional File 1: Table S3, Additional File 2: Table S4), TMB, MSI score (Additional File 1: Fig. S3), extent of genomic CNA (Fig. 2B, Additional File 1: Fig. S4), and mutational signatures (Additional File 1: Fig. S5). Four molecular subtypes were represented in the generated PDX models (Fig. 2A, Additional File 1: Fig. S6). One PDX model was *POLE*-mutated. It contained p.Pro286Arg *POLE* mutation in the exonuclease domain, previously reported in EC and shown to lead to a particularly strong mutator phenotype [31]. This PDX was characterized by an ultra-high TMB (>600 Mutations/Mb), a CNA stable genome, low MSI score (<3%), and a *POLE*-associated mutational signature, consistent with genomic profiles reported for *POLE*-mutated EC cases [3]. One PDX model was classified as CN-low p53 wild-type, since it lacked *TP53* or *POLE* mutations, had a low number of CNA segments, and was MSI-stable. It was characterized by a relatively low TMB, and moderately stable genome. Five PDX models were classified as MMRd, based on a high TMB (>20 Mutations/Mb), high MSI scores (≥3%), and MMRd-associated mutational signatures. One MMRd model (PDX12) had a significantly higher somatic mutation rate of >200 Mutations/Mb and was found to have two distinct MMR-associated mutational signatures compared with other MMRd models: Signature 20 and 14. Signature 14 is commonly found in cancers with ultra-high mutation rate and has been associated with concurrent MMRd and *POLE* mutations. A somatic *POLE* mutation p.Tyr956His (*n*=5 in COSMIC) was identified in PDX12, albeit not in the exonuclease domain.

Four PDX models were classified as CN-high/p53mut. This included PDX23 with mixed serous histology and three UCS models. PDX23 was characterized as *TP53* wild-type, whereas the UCS models all had *TP53* mutations. In the original TCGA study, 55/60 (92%) CN-high ECs carried mutations in *TP53* [3] and in the larger pan-cancer TCGA cohort, 142/163 (87%) CN-high ECs carried mutations in *TP53* [32]. Despite the difference in *TP53* mutation status, they all shared low TMB (≤10 Mutations/Mb), high degree of genomic instability (>25% of genome with CNAs and >15 CN segments), and low MSI scores, suggesting microsatellite stability. The UCS models had no dominant signature detected (<30% of somatic mutations attributed to a single signature; Additional File 1: Fig. S5), whereas the serous model had a dominant APOBEC signature.

Molecular subtyping of the other model with serous histology revealed it was MMRd (PDX53), which was consistent with a germline *MSH6* mutation (p.Tyr214*) in the patient and the finding that 13% of patients with germline MMR mutations have mixed serous histology [33]. Somatic frameshift mutations in *MSH6* were detected in three of five MMRd models, including PDX53, which also carried a germline *MSH6* mutation. Somatic mutations detected in the other genes were consistent with TCGA findings. Protein-altering somatic variants were commonly detected in *PTEN*, *ARID1A*, *PIK3CA*, *KRAS* and *RPL22* genes. All MMRd models contained somatic *PTEN* and *ARID1A* mutations, and four of five contained *RPL22*, most of which were inactivating frameshift or nonsense mutations (Fig. 2A, Additional File 2: Table S4). Mutations in *RPL22* occur at an A8 homopolymer and have been detected in 51/148 MSI ECs (35%, TCGA pan-cancer cBioportal analysis) [32], as well as 12/23 (52%) MSI ECs [34] and 17/34 MSI EC [35]. Recent functional studies suggest RPL22 acts as a haploinsufficient tumor suppressor involved in inducing senescence through CDK4 inhibition [36].

### Variable intra-tumor heterogeneity observed in MMRd PDX models

**Fig. 3:**
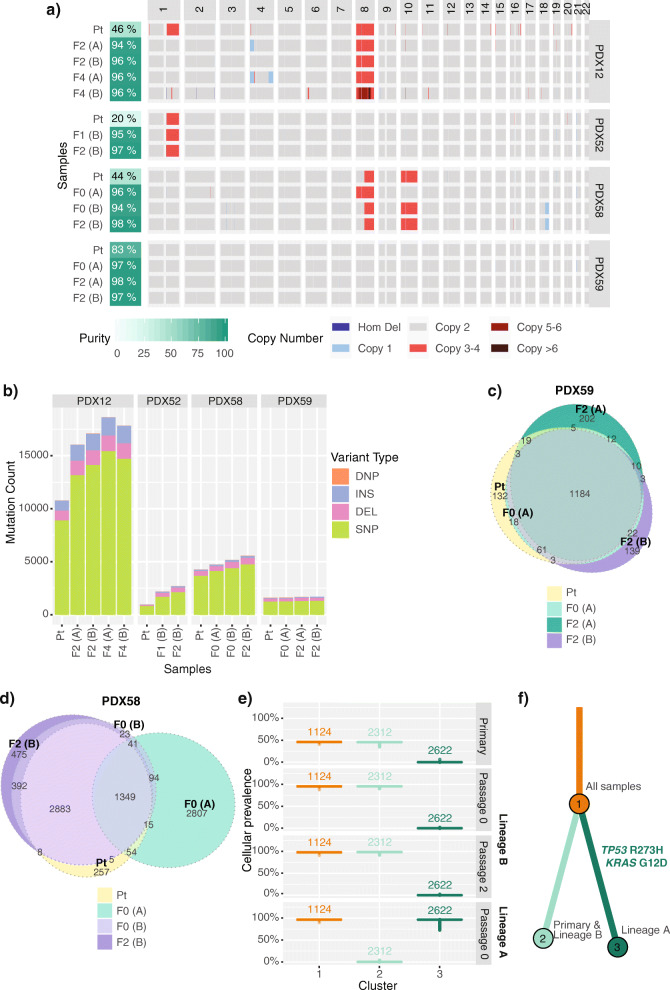
Intra-tumor heterogeneity observed in the MMRd EC PDX models. **A** Somatic genome-wide levels of CNA and **B** total somatic mutation count in the four MMRd models, where primary tumor sample was analyzed by WES and by SNP arrays. Tumor purity was estimated from the mode of somatic variant allele frequencies (Additional File 1: Fig. S10). Varying degrees of mutational heterogeneity visualized by Euler diagrams of somatic substitutions called by qBasepileup in **C** PDX59 and **D** PDX58 MMRd models. **E** Cellular prevalence and **F** the clonal evolution tree of the top three mutational clusters (with ≥5% of all somatic substitutions) detected in the PDX58 model by PyClone. Values shown above boxplots represent the number of substitutions contributing to each cluster. Length of branches is proportional to the number of substitutions attributed to that clone. Tumor samples are grouped by patient ID. PDX samples are labeled by passage number (F0—1^st^ transplant, F1—2^nd^ transplant, F2—3^rd^ transplant, etc.) and lineage in brackets (A, B). DEL, deletion; DNP, double nucleotide polymorphism; INS, insertion; SNP, single nucleotide polymorphism; Hom Del, homozygous deletion

To study the intra-tumor heterogeneity and to evaluate how well the PDX models recapitulate primary tumors, we focused in detail on four of five MMRd models (where matched primary tumor sample was available) as these might be expected to accumulate changes during passaging based on their defective DNA mismatch repair. We observed CNA and LOH differences in PDX12 and PDX58 (Fig. 3A, Additional File 1: Fig. S7a), but the overall genome-wide levels of CNA and LOH remained low in both primary and PDX tumor samples (Additional File 1: Fig. S7b). To investigate the distinct CNAs, we looked at additional PDX samples assessed with SNP arrays. For PDX12 model, we detected chr1q gain in primary tumor and lineage C PDX samples but not in lineages A or B, whereas lineage A PDX samples consistently had chr4p16-15.1 single copy loss that was not detected in the tested primary tumor or other PDX lineages (Additional File 1: Fig. S8). These lineage differences were observed from early passages (passage 1), suggestive of pre-existing subclonal events. We also detected chr4q31.3-35.2 single copy loss in the lineage A passage 4 sample that could have been PDX-acquired or in a rare pre-existing subclone. For PDX58, we similarly detected likely pre-existing subclonal events. Lineage A had distinct CNA profile from passage 0 compared to primary tumor and lineage B PDX (full chr8 gain and no chr8p LOH), whereas lineage B had the same CNA events as the primary tumor (Additional File 1: Fig. S9).

The TMB for primary tumor and PDX models was stable across different passages of PDX samples (passages 0–4); however, we observed a substantially higher number of mutations in PDX samples compared with matched primary samples in three PDX models (Fig. 3B). This was likely due to lower tumor purity observed in the primary samples compared with PDX samples (Additional File 1: Fig. S10). Indeed, PDX59 with the highest tumor purity (83% compared to 20–46% for other primary samples) had the most comparable number of somatic mutations to the matched PDX samples. For three of four models, 83–99% of the somatic substitutions in the primary tumor were also detected in all tested PDX samples, with only limited heterogeneity observed between different lineages of the PDX (Fig. 3C and Additional File 1: Fig. S11). In PDX58, however, we observed that one lineage of the established PDX shared only a third of its somatic substitutions with the primary tumor sample (Fig. 3D). A clonality analysis of PDX58 using PyClone identified two distinct mutational clones (PDX lineages A and B) that likely diverged early in the tumor evolution and were unintentionally selected during the initial tumor fragment transplantation (Fig. 3E, F). Mutations unique to lineage A included an activating *KRAS* mutation (p.Gly12Asp) and a hotspot *TP53* mutation (p.Arg273His). Mutations found only in primary tumor and lineage B included truncating *ARHGAP35* (p.Asn1028fs) and *ARID1A* mutations (p.Gly1848fs), albeit additional truncating *ARID1A* mutations were detected in lineage A sample and in all PDX58 samples (Additional File 1: Table S4). This clonality analysis was consistent with the distinct CNA profile detected in lineage A samples compared to the primary tumor and lineage B, which were more similar (Additional File 1: Fig. S9). Overall, since the greatest variability was observed between different lineages of the established PDX models and not between the passages, we concluded that this was likely due to spatial heterogeneity present in the original patient tumor.

For the PDX models where DNA from the primary tumor sample failed quality control for WES, we assessed the heterogeneity within the PDX samples. We observed minimal mutational heterogeneity in non-MMRd models (PDX24, PDX21 and PDX23) and some heterogeneity in MMRd model PDX53, particularly in two different lineages (Additional File 1: Fig. S12).

### Variable intra-tumor heterogeneity observed in p53mut uterine carcinosarcoma PDX models

**Fig. 4:**
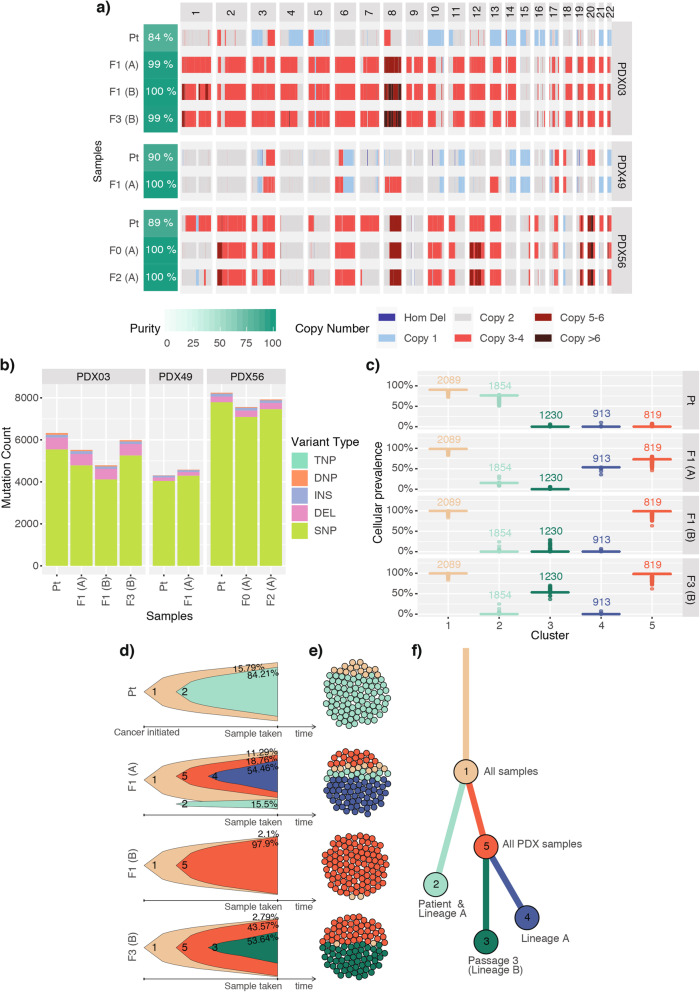
Intra-tumor heterogeneity and clonal evolution observed in p53mut UCS PDX models. **A** Somatic genome-wide levels of CNA and **B** total somatic mutation count in the three UCS models. Tumor purity was estimated by ascatNgs. **C** Cellular prevalence of the top five mutational clusters with ≥5% of all somatic substitutions detected in the PDX03 model by PyClone. Values shown above boxplots represent the number of substitutions contributing to each cluster. **D** Fish plots and **E** cellular population depictions of the top five mutational clusters detected in the PDX03 carcinosarcoma model. Percentages shown in the fish plots are the estimated proportions of cells containing that mutational cluster. **F** The clonal evolution tree inferred by ClonEvol, where length of branches is proportional to the number of substitutions attributed to that clone. Tumor samples are grouped by patient ID. PDX samples are labeled by passage number (F0—1^st^ transplant, F1—2^nd^ transplant, F2—3^rd^ transplant, etc.) and lineage in brackets (A, B). TNP, triple nucleotide polymorphism

To capture the genome instability and heterogeneity observed in the CN-high/p53mut UCS models, we performed WGS in three models to examine CNA changes in more detail. The overall genome-wide levels of CNA and LOH were comparable between the primary and matched PDX samples, with the exception of PDX03 model (Fig. 4A, Additional File 1: Fig. S13), which harbored a whole-genome duplication (WGD) not detected in the primary tumor. By examining CNA profiles of the additional PDX samples assessed by SNP arrays for models PDX49 and PDX56, we also observed a number of CNAs that appeared subclonal either in the primary sample or in the PDX samples. For PDX49 these included single copy loss of chr1p36 (primary tumor only), chr15 (subclonal in primary), chr21 (subclonal in passage 0 and enriched in passage 1), and chr22 and gain on chr8 (Additional File 1: Fig. S14), while for PDX56 these were gains on chr1, chr2, chr10, and chr12 (Additional File 1: Fig. S15). Importantly, we did not observe reoccurring CNAs that were enriched in PDX samples (i.e., PDX-passage specific) across the assessed models as had been previously reported for PDXs representing other tumor types [37]. The total number of somatic mutations detected across the whole genome for each UCS model was consistent between the primary tumor and the matched PDX samples, with no increase in mutation number detected with passaging (Fig. 4B).

For PDX03, which contained a WGD in the PDX and not the matched primary tumor, PyClone clonality analysis revealed a high degree of heterogeneity in the model, with five major different mutational clones detected (Fig. 4C-E) that were associated with multiple samples in PDX lineages A and B. Clone 2 was the predominant clone in the primary tumor sample (predicted in around 80% of tumor cells), but was detected at only around 15% in lineage A, and was absent in lineage B. Interestingly, using Battenberg we identified a CNA subclone in lineage A with a similar copy number profile to the primary tumor (Additional File 1: Fig. S16a-d). In support of this, the ploidy estimated from SNP array analysis of the additional PDX samples identified two early lineage samples (passage 0 and 1) from lineage A that had estimated ploidy of 2, same as the primary tumor sample (Additional File 1: Fig. S16e). We therefore concluded that the WGD event was already present in a subclone of the primary tumor, although not detected in the sample taken for WGS analysis. In the other two carcinosarcoma models, clonality analysis also revealed heterogeneity, although not to the same extent as seen in the PDX03 model (Additional File 1: Fig. S17-18).

### Assessment of PARPi responses and HR status in PDX models

Since the established PDX models were found to reflect the primary tumors, we evaluated their use for testing molecularly-targeted treatments. Potential therapeutic options for all 11 PDX models were identified using their genomic profiles and Cancer Genome Interpreter analysis (Additional File 1: Fig. S19). Multiple therapeutic options were identified, with three or more options detected per PDX model. PARP, mTOR, and PI3K pathway inhibitors, as well as PD1 inhibitors, were identified among the most common potential treatment options. A large number of therapeutic agents were identified for tumors with a high TMB (POLE/MMRd); thus, functional testing is required to determine whether these models are responsive, given many of the mutations could be passenger events.

**Fig. 5:**
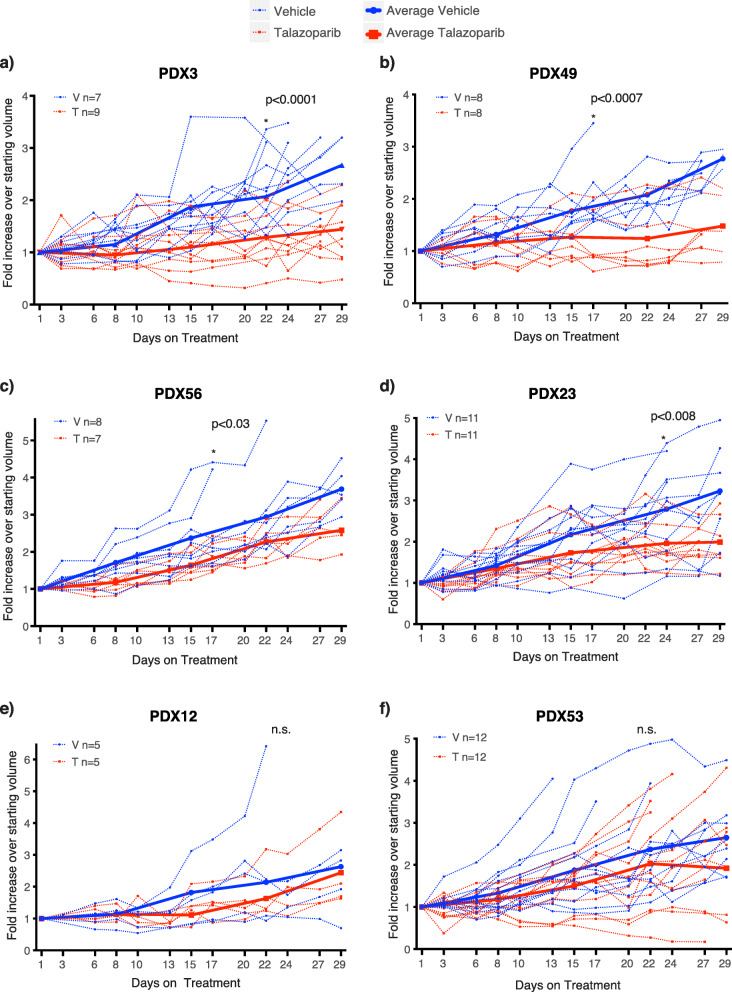
Talazoparib responses in EC and UCS PDX models. Talazoparib responses in **A** PDX03 — CN-high/p53mut UCS; **B** PDX49 — CN-high/p53mut UCS; **C** PDX56 — CN-high/p53mut UCS with somatic *ARID1A* deletion; **D** PDX23 — CN-high EC; **E** PDX12 — MMRd EC with somatic *PTEN*, *BRCA2*, *ATM*, and *PALB2* mutations; **F** PDX53 — MMRd EC with somatic *PTEN*, *ATM*, *BRCA1*, and *MRE11A* mutations. Recipient mice bearing PDX at starting volume of ~150–350 mm^3^ were randomized to treatment with vehicle or talazoparib (0.33mg/kg) for 28 days (6 days on, one day off) via oral gavage. Analysis for significance between treatment groups was performed using a repeated mixed effects analysis (which can account for random missing measurements) on the day the first mouse was sacrificed based on tumor size (e.g., 17, 22, and 24 days), except for PDX53 where 2 vehicle mice were sacrificed early and excluded. n.s, not significant; *, significant difference (*p-*value shown)

PARPi has been previously identified as a promising therapeutic strategy for EC. Therefore, we evaluated PARPi responses in a subset of the established PDX models. Highly potent PARPi talazoparib was selected due to its significantly higher PARP trapping ability [38] and was used to treat the four CN-high PDX models and two MMRd models in vivo (Fig. 5). PDX03 and PDX49 showed the most sensitivity, with average tumor growth inhibition (TGI) showing the equivalent of stable disease by Response Evaluation Criteria In Solid Tumors (RECIST). PDX56 and PDX23 showed significant TGI, albeit this would translate to progressive disease by RECIST. No effect of talazoparib was seen in the MMRd models with multiple heterozygous missense mutations and/or LOH in DNA repair genes (PDX12 and PDX53). Interestingly, the in vivo validation of in silico PARPi response predictions was quite poor. Only two of six PDX models were predicted to respond correctly—PDX23 was predicted to respond and showed TGI in vivo, and PDX12 was predicted to not respond.

**Fig. 6:**
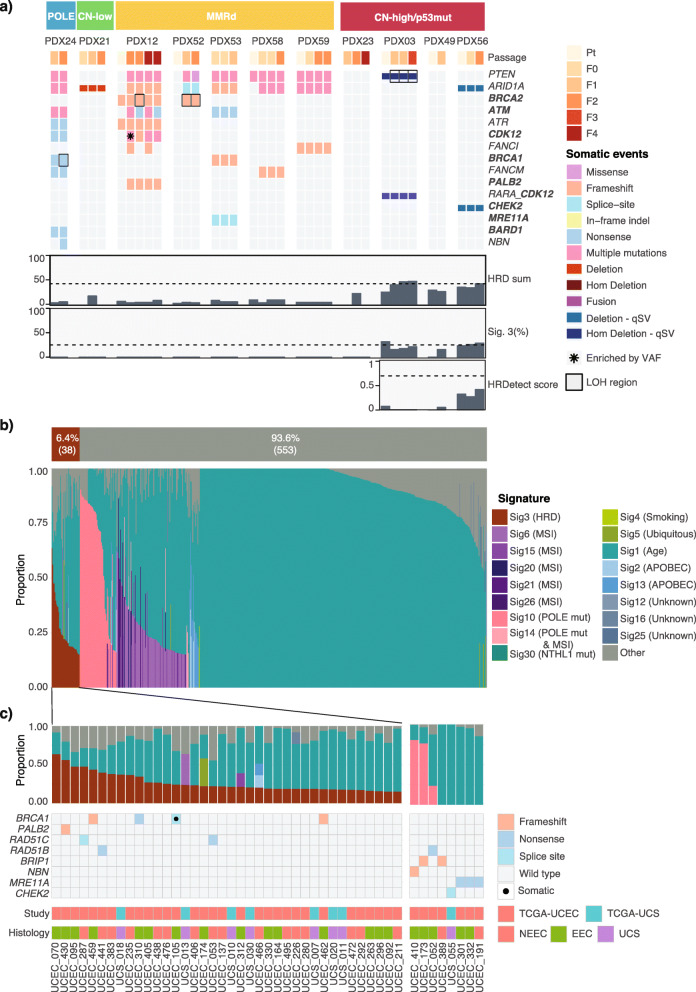
Genomic HRD assessment in EC PDX models and public data**. A** HRD assessment in PDX models. Somatic substitutions, indels, CNAs and SVs are shown for DNA repair related genes, including *PTEN* and *ARID1A* (Additional File 1: Table S5, Additional File 2: Table S6). HR-related genes are highlighted in bold. No pathogenic or likely pathogenic germline substitution and indel variants in these genes were detected. HRD sum scores were determined using scarHRD from SNP arrays and WGS data, where available (unable to calculate scores for PDX21 F1 and F2 samples and PDX23 F1 and F4 samples due to noisy arrays). Percentage of Signature 3 was determined with deconstructSigs using COSMIC v2 signatures. Only WGS data is shown for PDX03 and PDX49, where WES and WGS was performed. HRDetect scores were determined only for samples with WGS data. **B** Mutational signature assignment for TCGA-UCEC and TCGA-UCS cohorts (*n*=591). Signature assignment was performed using deconstructSigs with 15% minimum signature cut-off. **c** TCGA-UCEC and TCGA-UCS cases with possible HRD. Cases with pathogenic or likely pathogenic variants in HR-related genes (Additional File 1: Table S5, Additional File 2: Table S7) or cases with Signature 3 detected are included

Due to the limited responses to single agent PARPi observed in vivo, we characterized the PDX HR status in detail. We firstly estimated the HRD scores for the primary and matched PDX samples with WGS or SNP array-estimated CNA data. All of the primary tumor samples had HRD scores below the threshold of ≥42, a previously defined cut-off for HRD breast and ovarian cancers [39, 40]. Two of three CN-high carcinosarcoma models had some F1 or F2 PDX samples with HRD scores just over the threshold (Fig. 6A).

We then assessed the mutational signatures, specifically contribution of COSMIC Signature 3, an alternative marker of HRD. Three CN-high carcinosarcoma models had low mutational contribution of Signature 3, between 20 and 30%, while the other models had no Signature 3 contribution (Fig. 6A). However, rearrangement signature analysis on the three carcinosarcoma models did not detect *BRCA1/2-*associated signatures (Additional File 1: Fig. S20). For carcinosarcomas that were sequenced with WGS, we also utilized a weighed combinatorial approach for HRD prediction, HRDetect [28], which takes into consideration mutational and structural variant signatures, microhomology indels, and HRD scores described above. All three models had HRDetect probability scores well below the HRD threshold of 0.7, established and tested in breast, ovarian, and pancreatic cancer (mean scores of 0.02, 0.03, and 0.34 for PDX03, PDX49, and PDX56, respectively) [28]. Finally, we looked for the presence of pathogenic or presumed pathogenic germline and somatic mutations in the HR-associated genes (Additional File 1: Table S5) and somatic mutations in *PTEN* and *ARID1A*, since mutations in these genes have been associated with PARPi sensitivity in the pre-clinical setting [10, 13]. Apart from recurrent presumed pathogenic mutations in *PTEN* and *ARID1A*, we did not detect any clear driver mutations in HR-genes with evidence of enrichment or LOH in CN-high/p53mut models. We did detect a large number of likely passenger mutations in hypermutated models (MMRd/ POLE; Fig. 6A; Additional File 2: Table S6). The genomic characterization and the lack of in vivo tumor regressions in response to PARPi suggested that all models were HR proficient.

### HR deficiency is predicted to be a rare event in UCEC and UCS

Since we did not observe clear evidence of HRD in the PDX models, we wanted to estimate the rate of HRD in a larger cohort of unselected UCEC and UCS patients. We assessed HR status in the expanded TCGA cohort of 534 endometrial carcinomas (TCGA-UCEC: *n*=393 endometrioid EC and *n*=141 non-endometrioid EC) and 57 uterine carcinosarcomas (TCGA-UCS). De novo detection of mutational signatures identified six signatures associated with age, APOBEC, MMR, and POLE, but not HRD-associated Signature 3 in either of the datasets (Additional File 1: Fig. S21). We then applied the same approach that was used for the PDX models — assigning known COSMIC signatures (including HRD Signature 3) to the mutational profiles of each sample. Using this approach, we predicted that only 6.4% (38 of 591) of all analyzed samples had Signature 3 detected, using 15% minimum signature contribution cut-off to avoid overfitting (Fig. 6B). Signature 3 was detected in 11.4% (16/141) of non-endometrioid EC, 3.8% (15/393) of endometrioid EC and 12.3% (7/57) of UCS cases.

We also looked for germline and somatic variants in HR genes in these 591 samples (Fig. 6C, Additional File 2: Table S7). Only 2.5% (15/591) cases were found to harbor germline pathogenic or presumed pathogenic HR gene variants: 3.5% (5/141) of non-endometrioid, 2.3% (9/393) of endometrioid, and 1.8% (1/57) of UCS cases. Seven variants in *BRCA1*, *PALB2*, *RAD51C*, and *RAD51D* genes were concurrent with the presence of mutational Signature 3, whereas the variants in *BRIP1*, *NBN*, *MRE11A*, and *CHEK2*, as well as one variant in *RAD51B*, were detected in cases without Signature 3. This finding was in line with a previous report in breast cancer, where mutations in DNA-damage signaling pathway genes, such as *ATM* and *CHEK2*, were not associated with increased Signature 3 [41]. Somatic mutation analysis was restricted to 38 cases with detected Signature 3. Only one somatic pathogenic variant in *BRCA1* was detected in endometrioid cancer.

## Discussion

In this study, we aimed to generate and characterize multiple PDX models of EC to assess their suitability as pre-clinical models for studying the disease. The highest engraftment rate was achieved for tumors of grade 2 or 3 histology and fresh tumor material (15/25; 60%). This is comparable with the 60% engraftment rate previously reported for EC [42] and 62% for leiomyosarcomas and carcinosarcomas [43]. Engraftment was less successful after storage at −80 °C or overnight at 4 °C. We also observed an incidence of lymphoma formation (13%) similar to that previously reported in ovarian cancer (11%), which in future studies can be reduced by the addition of rituximab during implantation [44]. The proportion of the molecular subtypes in this study differs from that seen in primary tumors reported by TCGA [3], as the process of PDX engraftment selects for more aggressive subtypes. Little data exists on the frequency of these subtypes in metastatic EC; however long-term follow-up is available for several large population-based cohorts. As expected, the proportion of patients with the p53mut EC subtype increases between patients diagnosed with low/intermediate risk EC (9%) [2] and those diagnosed with high risk EC (23%) [45]. Similarly, in the combined ProMisE cohorts the p53mut subtype comprises 18% of patients initially diagnosed but 48% of patients that die due to EC [4–6]. Although the proportion of MSI cases remains similar between patients diagnosed and those that die of EC (23%), in the combined ProMisE cohorts, the CN-low ECs comprise 47% of those diagnosed, but only 22% of EC specific deaths [4–6]. This explains in part the number of CN-high/p53mut and MMRd PDX models compared to POLE and CN-low PDX models in our cohort.

To understand the suitability of pre-clinical PDXs models to study EC, we undertook an in-depth genomic characterization of patient primary and matched serial PDX tumor samples of EC. Immunohistochemistry studies in small panels of EC PDX models have been reported previously [46, 47]; however, no molecular or genomic analysis of these models was included. The Amant laboratory has reported some molecular characterization of EC PDXs (e.g., microsatellite stability versus instability) with WES reported for four models [42]. They have also reported copy number changes in two PDXs from one lineage in a panel of sarcomas and carcinosarcomas with WES reported in one model [43]. To the best of our knowledge, our report is the first to describe de novo mutational signature and copy number analysis across a panel of EC PDXs. The established PDX models recapitulated key morphological and genomic features present in the molecular subtypes [3]. Interestingly, no distinct PDX-specific mutational signatures were found using de novo signature analysis, and the overall mutational profiles were very similar between the primary and matched PDX samples. We also did not observe an accumulation of PDX-specific CNA events in these PDX models, as was previously reported in PDX models of breast, brain, lung, colon, and pancreatic cancers [37]. In keeping with the earlier study [37], we observed that the CNA dynamics were likely driven by the selection of pre-existing subclones, as most of the CNA differences were observed between PDX lineages and not between passages. Taken together, these results suggest that recurring PDX-specific evolution is minimal in these models, and these models reliably represent the driver events and molecular subtypes of the primary tumors.

### Intra-tumor heterogeneity

EC tumors are composed of multiple complex sub-clonal cell populations resulting in intra-tumor heterogeneity [48, 49]. Maximum tolerated dose chemotherapy regimens aim to eradicate the entire tumor but rarely achieve it, often leaving resistant sub-clones that possess a growth advantage and are free to expand. Hence, genomic intra-tumor heterogeneity has clinical implications for EC and needs to be characterized to enable precision medicine. In this study, we transplanted undisturbed tumor fragments rather than minced tumor cells which allowed us to observe great variation in the intra-tumor heterogeneity among different PDX lineages and passages.

In two of four models, where multiple lineages were sequenced, we detected high levels of intra-tumor heterogeneity that could have a potential impact on treatment responses. The greatest variability was observed between the different lineages (detected in early passages) and not between the passages, indicating that this heterogeneity was pre-existing in the primary tumor, although it is unclear whether the subclones were selected due to chance or due to a selective advantage in the PDX. In the absence of much deeper sequencing for detection of rare subclones, we were unable to determine the full degree of intra-tumor heterogeneity in the primary tumor samples due to lower tumor purity and different tissue fragments used for the initial transplant and DNA extraction. In the MMR-deficient model (PDX58), one of the established lineages was enriched for a subclone with hotspot *KRAS* and *TP53* mutations and over 50% private mutations, suggesting that this subclone had diverged early on in the tumor evolution. *KRAS* mutations have been linked to drug resistance in multiple cancers [50, 51], and *TP53* mutations are associated with poor prognosis in EC [3]. In the *TP53*-mutant UCS model (PDX03), one of the lineages had a WGD event together with other subclonal mutations. WGDs are frequently detected in UCS (90%) [9] compared with epithelial EC, as well as in metastatic cancers across multiple cancer types [52] and have been associated with poor prognosis [53]. PDX models that capture pre-existing intra-tumor heterogeneity such as described here make a perfect tool for studying the effects of individual genomic events on tumor evolution, progression, and drug responses and should be explored further. However, the extensive spatial heterogeneity reported here can also result in challenges for predicting treatment responses based on molecular profiling of clinical tumor samples, as typically only a small tumor fragment is sequenced and key mutations can be missed. As such, liquid biopsies or representative sequencing techniques [54] may provide less biased molecular profiling results for recommending patient treatment options.

### PARP inhibitors in EC

PARPi sensitivity has previously been reported in EC, although to date the work has been performed in cell lines, which do not faithfully represent all of the EC molecular subtypes [10, 12]. The proposed biomarkers of PARPi response in EC are diverse and include *PTEN*, *ARID1A* or *MRE11A* loss [10, 12, 13], *TP53* mutations, and cumulative effect of multiple somatic hits in HR genes in hypermutated MMRd EC [55]. Our PDX EC cohort had a representation of all of these events, so we could investigate their effect on PARPi response in vivo. By performing HRD scarring and mutational signature assessment, commonly used for classifying HRD-ness in ovarian and breast cancers [56], we determined that our EC models were all likely HR-proficient. Interestingly, three UCS models with mutated *TP53* had intermediate HRD scores and some of the somatic mutations were attributed to Signature 3, although the HRDetect scores were well below the HRD threshold. Two UCS models showed disease stabilization in response to the potent PARPi talazoparib in vivo, whereas the CN-high serous and third UCS model showed significant TGI. This observation is consistent with an earlier study of PARPi niraparib in ovarian *TP53*-mutant PDX models, where some HR competent models showed disease stabilization or TGI, while HRD models showed much more pronounced responses with tumor regression [57]. The PDX models representing MMRd ECs were chosen as they contained damaging mutations in canonical HR genes and mutations in *PTEN* and *ARID1A*; however, talazoparib had no effect on TGI. It should be noted that although these PDX models harbored multiple damaging mutations in canonical HR genes none showed consistent enrichment in tumors. This data indicates that loss of PTEN or ARID1A or multiple heterozygous mutations in HR genes in MMRd ECs are not associated with response to PARPi as proposed [58].

It has been recently reported that up to half of non-endometrioid EC (predominantly p53mut) can harbor BRCA-associated genomic scars compared with only 12% of endometrioid ECs (CN-low/MMRd) [15]. Since our PDX cohort did not have a large representation of non-endometrioid EC (five of 11 models), it was possible that we missed the HRD cases by chance. However, our exploration of TCGA UCEC and UCS datasets also showed that HRD is likely rare in EC. We saw much lower rates of Signature 3, both in general (6.8%), and in the non-endometrioid cancers (12%) compared to the previous report [15]. This was likely due to a more conservative signature assignment approach used in our study. The mutational signature analysis approach can have a great influence on the identification of signatures, especially signatures with flat profiles (Signature 3, 5, and 8) [59]. Our rates of Signature 3 were more in line with a recent analysis of a smaller cohort of TCGA UCEC and UCS cases [60]. Furthermore, the rates of damaging mutations in HR genes were quite rare (1.6–3.6% depending on the histological subtype), consistent with another study looking at bi-allelic alterations in HR genes [14]. In this study, we focused on somatic HR mutations that were predicted to be damaging and only in cases with detected Signature 3, to avoid inclusion of possible passenger mutations in hypermutated MMRd and POLE cases. These factors could explain why we detected lower rates of HR mutations in EC compared to some other reports [61, 62].

The lack of tumor regressions in our EC PDX models in response to PARPi talazoparib and the infrequent HRD events in EC public datasets indicate that PARPi may not be sufficient as a single agent therapy in an unselected EC patient population. Nonetheless, PARPi may still have an important role to play in the management of EC and should be further investigated in combination with other treatments. Several PARPis including talazoparib have been shown to have strong PARP trapping effect [38], leading to replication stalling. This opens up the possibility to combine PARPi with other therapies for an enhanced anti-tumor activity, including cell cycle checkpoint inhibition, PI3K pathway inhibition, RNA Pol1 inhibition [63], or ICIs. Genomically characterized patient-derived organoids [64, 65] or explant models [66] would be more time and cost efficient than PDX models for assessing which PARPi combination/s have the best efficacy in a panel of CN-high EC. Genomically characterized PDX models, such as ours, will also be useful to perform pre-clinical assessment of PARPi and immunotherapy combinations in humanized PDX models, as performed in other cancers [67], to support the design of new clinical trials.

The limitations of this study include the absence of primary tumor samples for four models limiting the intra-tumor heterogeneity studies that could be performed, in vivo talazoparib responses for only six models, and a lack of in vivo sensitivity data for additional anti-cancer agents implicated by the Cancer Genome Interpreter data. In the future, more detailed studies on intra-tumor heterogeneity could employ single cell analysis.

## Conclusions

Although some EC subtypes are represented by only one to three PDX models, somewhat limiting the conclusions that can be drawn, we have shown that EC PDX models can capture intra-tumor heterogeneity, which should be accounted for and explored to improve treatment responses and patient outcomes. By combining genomic characterization and in vivo treatments, we also showed that PARPi talazoparib had some disease stabilization activity in CN-high/p53mut EC, which can potentially be enhanced by combination therapies.

## Supplementary Information

**Additional file 1: Supplementary Methods, Supplementary Tables 1-3,5 and Supplementary Figures.**

**Additional file 2: Supplementary Tables 4, 6-8** (descriptions provided in the file).

## Data Availability

The datasets (raw data files for WGS, WES, and SNP arrays) supporting the conclusions of this article are available in the European-Genome Phenome Archive under study number EGAS00001004666 at https://ega-archive.org/studies/EGAS00001004666 [68]. TCGA-UCEC data were downloaded from GDC data portal in October 2016, accessed through dbGaP accession number phs000178.v11.p8. TCGA-UCS data were downloaded from GDC data portal in March 2019. PDX models are available upon request with appropriate material transfer agreements and ethics approval*.* The code required to reproduce the figures in this manuscript can be found on https://github.com/okon/EC_PDX_genomics [69]. The code used to perform alignment to human and mouse genome references is available on https://github.com/ampatchlab/nf-pdx [70]. The in-house tools are available on https://github.com/AdamaJava/adamajava [71].

## Abbreviations

CNA: Copy number alteration
CN-low: Copy number low
CN-high: Copy number high
EC: Endometrial cancer
FFPE: Formalin-fixed paraffin-embedded
H&E: Hematoxylin and eosin
HR: Homologous recombination
HRD: HR deficiency
ICI: Immune checkpoint inhibitor
LOH: Loss of heterozygosity
MMRd: Mismatch repair deficient
MSI: Microsatellite instability
NSG: Nod scid gamma
p53mut: *TP53* mutant
PARPi: PARP inhibitor
PDX: Patient-derived xenograft
RECIST: Response Evaluation Criteria In Solid Tumors
SV: Structural variant
TCGA: The Cancer Genome Atlas
TGI: Tumor growth inhibition
TMB: Tumor mutation burden
UCEC: Uterine corpus endometrial carcinoma
UCS: Uterine carcinosarcoma
WES: Whole-exome sequencing
WGD: Whole-genome duplication
WGS: Whole-genome sequencing
